# Dry season limnological conditions and basin geology exhibit complex relationships with δ^13^C and δ^15^N of carbon sources in four Neotropical floodplains

**DOI:** 10.1371/journal.pone.0174499

**Published:** 2017-03-30

**Authors:** Gustavo H. Zaia Alves, David J. Hoeinghaus, Gislaine I. Manetta, Evanilde Benedito

**Affiliations:** 1 Programa de Pós-Graduação em Ecologia de Ambientes Aquáticos Continentais (PEA), Universidade Estadual de Maringá, Maringá, Paraná, Brasil; 2 Postdoctoral fellowship, National Council for Scientific and Technological Development (CNPq), Brasília, Distrito Federal, Brasil; 3 Department of Biological Sciences and the Advanced Environmental Research Institute, University of North Texas, Denton, Texas, United States of America; 4 Programa de Pós-Graduação em Biologia Comparada (PGB), Universidade Estadual de Maringá, Maringá, Paraná, Brasil; University of Hyogo, JAPAN

## Abstract

Studies in freshwater ecosystems are seeking to improve understanding of carbon flow in food webs and stable isotopes have been influential in this work. However, variation in isotopic values of basal production sources could either be an asset or a hindrance depending on study objectives. We assessed the potential for basin geology and local limnological conditions to predict stable carbon and nitrogen isotope values of six carbon sources at multiple locations in four Neotropical floodplain ecosystems (Paraná, Pantanal, Araguaia, and Amazon). Limnological conditions exhibited greater variation within than among systems. δ^15^N differed among basins for most carbon sources, but δ^13^C did not (though high within-basin variability for periphyton, phytoplankton and particulate organic carbon was observed). Although δ^13^C and δ^15^N values exhibited significant correlations with some limnological factors within and among basins, those relationships differed among carbon sources. Regression trees for both carbon and nitrogen isotopes for all sources depicted complex and in some cases nested relationships, and only very limited similarity was observed among trees for different carbon sources. Although limnological conditions predicted variation in isotope values of carbon sources, we suggest the resulting models were too complex to enable mathematical corrections of source isotope values among sites based on these parameters. The importance of local conditions in determining variation in source isotope values suggest that isotopes may be useful for examining habitat use, dispersal and patch dynamics within heterogeneous floodplain ecosystems, but spatial variability in isotope values needs to be explicitly considered when testing ecosystem models of carbon flow in these systems.

## Introduction

Recent studies in freshwater ecosystems are seeking to improve understanding of carbon flow in food webs by testing general conceptual models such as the River Continuum Concept (RCC; [1]), Flood Pulse Concept (FPC; [2]), Riverine Productivity Model (RPM; [3]), Riverine Ecosystem Synthesis [4] and River Wave Concept (RWC; [5]). Stable isotopes, particularly of carbon and nitrogen, have been influential in this work as natural tracers of energy sources and trophic interactions [6–14]. One major issue with testing the aforementioned models using stable isotope analyses (SIA) is the ability of the isotopes to reliably distinguish among potential autotrophic carbon sources and/or size fractions within and among ecosystems. This is often complicated by the diverse suite of potential carbon sources (both autochthonous and allocthonous) in freshwater ecosystems, and the spatially and temporally dynamic nature of carbon source relative abundances (e.g. [15–18]).

In freshwater ecosystems, the substrate for autochthonous photosynthesis is dissolved carbon dioxide or bicarbonate (grouped as dissolved inorganic carbon—DIC). The dominant form of DIC present in aquatic ecosystems is determined largely by pH [19, 20] which also influences the ratio of heavy to light isotopes of carbon (δ^13^C) of the DIC [21]. The δ^13^C of the DIC can also be affected by salinity or nutrient limitation [22, 23], pressure of CO_2_ as a result of terrestrial respiration of organic material [24], ecosystem area and metabolism [21, 25], lithology and hydrology [26], and basin geochemistry [27]. Subsequently, the factors that determine the δ^13^C of aquatic primary producers are complex because of the influences of spatial heterogeneity at multiple scales, such as local habitat, reach, watershed, hydrology and geochemistry [7, 14, 28]. Opposite to strictly aquatic primary producers (i.e. algae, periphyton), macrophytes and riparian plants utilize atmospheric CO_2_ as the photosynthetic substrate, which they assimilate via leaf stomatal absorption [29]. The δ^13^C values of these plants are mainly influenced by its photosynthetic pathway (i.e. C_3_ or C_4_; [30]), however, under stressful conditions (i.e. nutrient limitation for macrophytes or water limitation for riparian plants), they can close their stomata, which leads to a lesser enzymatic discrimination against ^13^CO_2_ and increasing tissue δ^13^C values [31].

Similarly, δ^15^N of basal carbon sources can vary according to many factors, including salinity, basin geochemistry, level of eutrophication and preference for the form of dissolved inorganic nitrogen by plants (NH_4_^+^ or NO_3_^-^; [32–35]). Pollution from urban sewage or agriculture is an important factor affecting δ^15^N [36–40], and areas with human wastewater inputs are consistent with high amounts of dissolved inorganic nitrogen and elevated δ^15^N in the sediment and organisms [32, 40, 41]. For this reason, nitrogen stable isotope composition is a useful tracer of biogeochemical processes in the water column and of nutrients derived from multiple sources (e.g. animal wastes, septic systems, sewage treatment plants; [40, 42–45]).

Tropical river floodplain ecosystems are well-suited for examining factors affecting stable isotope values of basal carbon sources. Specifically, tropical river floodplains are characterized by high species and functional diversity of carbon sources and consumers, spatial heterogeneity, importance for freshwater biodiversity, and historical use in testing river ecosystem concepts (e.g. [8, 16]). Furthermore, evidence from previous studies in floodplain ecosystems (e.g. [6, 27, 46, 47]) helped to frame the discussion of potential drivers of variability in isotopic values of sources. For example, Jepsen and Winemiller ([47]) found that basin geochemistry (i.e. whitewater vs. blackwater) determined between-river isotopic differences in sources and consumers in tropical rivers of Venezuela, allowing for isotope values to be used as tracers of fish movement between systems [48, 49]. Understanding the factors that affect variability in stable isotope values of sources within and among floodplains is important for tests of the aforementioned river ecosystem concepts. If local limnological conditions distinguish source values within floodplains in a predictable manner, those differences may 1) yield a spatially and seasonally relevant isotopic landscape or “isoscape” enabling stable isotopes to be used as tracers of organism movement among patches within floodplains (e.g. Brosi *et al*. ([50]) for bees in a fragmented tropical landscape, and Fry *et al*. ([51]) for fishes moving from marshes to offshore in a freshwater Florida lake), or 2) be used to align baseline values for comparisons within and among systems in a manner analogous to lipid normalization models that correct for lipid concentration effects on isotope values in consumer tissues (e.g. [52, 53]).

In this study, we quantified local limnological conditions and assessed variation in stable carbon and nitrogen isotope values of carbon sources at multiple locations in four Neotropical floodplain river systems. Six carbon sources are analyzed, but we primarily focus on periphyton, phytoplankton and particulate organic carbon as stable isotope ratios of those sources are expected to reflect effects of limnological conditions over a timescale (i.e. hours to days) more suitable for comparison with our field surveys. The Paraná, Pantanal, Araguaia and Amazon, study systems capture continental-scale differences in geological formations as well as include significant within-basin heterogeneity of local limnological conditions. Using the aforementioned dataset, we ask the following questions: 1) Can geological formation (i.e. basin identity) and local limnological conditions (e.g. pH, turbidity, nutrient concentrations) predict among- and within-floodplain differences in δ^13^C and δ^15^N of carbon sources?, and 2) What is the relative importance of geology versus local limnology in determining variation in isotopic values of carbon sources?

## Material and methods

### Ethics statement

All samples were properly collected with all required permissions from the Brazilian Environmental Ministry (Instituto Chico Mendes de Conservação da Biodiversidade (ICMBio), Sistema de autorização e informação em Biodiversidade (SISBIO)), under protocol number 29652.

### Study systems and river classifications

**Fig 1:**
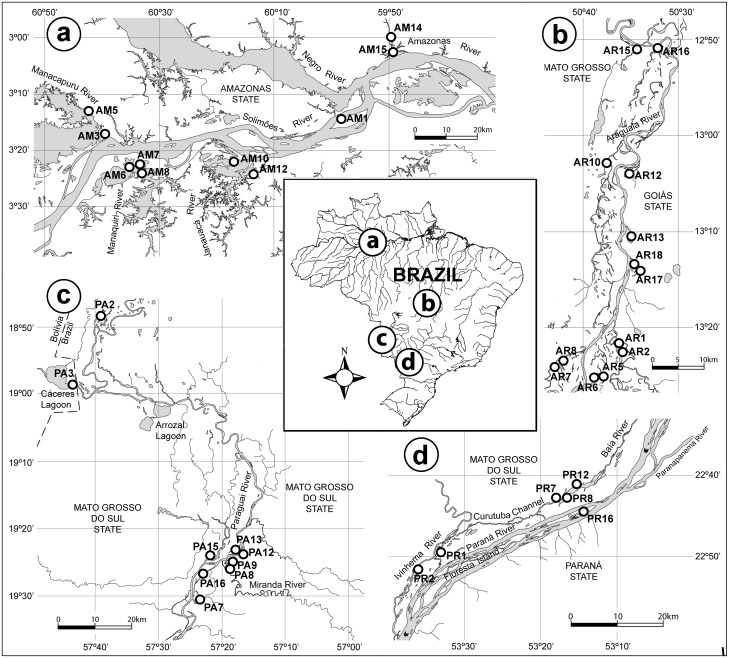
Map of the sampling locations in each basin study system. (i.e. **a** = Amazon floodplain; **b** = Araguaia floodplain; **c** = Pantanal floodplain; **d** = Paraná floodplain). Each point in the map represents a lake where carbon sources were sampled and limnological parameters were quantified. AM = Amazon; AR = Araguaia; PA = Pantanal; PR = Paraná. This figure is similar but not identical to the original image (i.e. in Arrieira et al. [56]), and is therefore for illustrative purposes only.

This study was conducted in four Brazilian river-floodplain ecosystems: Upper Paraná River floodplain (Paraná, Baía and Ivinheima rivers), Pantanal floodplain (Paraguai and Miranda rivers), Araguaia River floodplain and Amazon floodplain (Solimões and Amazonas rivers) (Fig 1). All of these systems have regular flooding periods during the rainy season of the Neotropics [2], and support high biodiversity of organisms, including autotrophic carbon sources. Rivers can be generally classified as whitewater, clearwater or blackwater [54], based on conditions in the catchment area that affect water color, load of suspended solids, pH, and load of dissolved minerals. Applied to our study systems, only the Solimões/Amazonas is whitewater [55], and the remainder may be primarily considered clearwater (see descriptions of the ecosystems below). That being said, Sioli’s ([54]) classification of large clearwater rivers includes pH values ranging between 6 and 6.7, and combined with the distribution of clearwater rivers over diverse geological zones, demonstrates that ‘clearwater’ is a chemically (and biologically) heterogeneous classification with only a poorness in suspended particles as a common character [54]. Furthermore, floodplain ecosystems have a wide range of the aforementioned parameters due to their inherent spatial heterogeneity (e.g. among channels and floodplain lakes of various size and connectivity), and not all location environments fit nicely into the same general classification applied at the landscape scale.

The Upper Paraná River is extensively impounded, with over 130 major reservoirs (dam height ≥ 10 m) [57, 58] that modify hydrology and retain sediment and nutrients, resulting in clear oligotrophic water in the main channel of the Paraná [59]. The floodplain is a mosaic of lakes and channels belonging to the Paraná and two primary tributaries (Baía and Ivinheima rivers). The base of the floodplain is Cretaceous sandstone overlain by gravelly, sandy, colluvial and alluvial unconsolidated deposits [60]. Watercolor ranges from clear to greenish to light brown, pH is 6.16 to 6.93, and watersheds are comprised by a mix of forest, pasture and urban areas.

The Pantanal is one of the world’s largest tropical wetlands, occupying approximately 140,000 km^2^. The flood pulse in the northern region coincides with the rainy season but there is a time lag before flooding in the southern region as water flows southward through the main tributary (Paraguai River), various streams and non-channelized flow paths [61,62,63]. Coarse silty-loam irregular deposits form the lower plain of the Pantanal geologic formation [64,65]. Watercolor across the sampled region ranges between green and brown, with abundant aquatic vegetation, pH 6.04 to 7.44, and a primarily grassland and forested watershed.

The Araguaia River in the eastern Amazon drains an area of 320,290 km^2^, including the middle Araguaia which is accompanied by a well-developed alluvial plain [66]. This plain is considered a complex mosaic of morpho-sedimentary units and the basin is dominated by rocks of various geological ages and formations and sedimentary deposits [66,67]. Approximately 76% of the Araguaia watershed is savannah (locally known as *Cerrado*). The Cerrado ecoregion is considered a hotspot for biodiversity [68] and is the headwater region of the major rivers of eastern South America. Rivers located in the Cerrado are usually classified as clearwater, and our study locations had light brown to greenish water color and pH from 6.33 to 7.37.

Wetlands on the alluvial floodplains of the Amazon River and tributaries in Brazil cover over 300,000 km^2^ [69]. The Brazilian Amazon floodplain is comprised by the Negro and Solimões Rivers that join to form the Amazonas River. The geologic basin consists of sandstones, siltstones, intercalated lignites and clay conglomerates [70]. The Solimões and Amazonas are classic examples of Amazonian whitewater rivers, with nutrient rich, pH neutral (6.2–7.2), turbid water due to suspended mineral solids primarily originated from the Andes Mountains [54, 71]. Limnological conditions among floodplain lakes in our study (some of which were isolated from the main channel) were heterogeneous, including a range of pH from 5.02 to 8.83.

### Sampling

Sampling was conducted in floodplain lakes during the late dry season for each floodplain (i.e. September 2011 for Paraná, March 2012 for Pantanal, November 2011 for Araguaia and October 2011 for Amazon). Samples were collected during the late dry season to incorporate the highest degree of heterogeneity in floodplain limnological conditions [72], which are expected to affect stable isotope values of carbon sources. Temperature, dissolved oxygen concentration, electrical conductivity, turbidity, pH and transparency were measured using handheld probes (YSI 550A, Digimed DM-2) and a 25-cm Secchi disk at each location in each basin (i.e. six in Paraná, nine in Pantanal, 13 in Araguaia and 10 in Amazon; Fig 1). Water samples were collected from the subsurface limnetic region for laboratory analysis of phosphorous and nitrogen concentrations (Table 1).

**Table 1:** Mean (±standard deviation) of limnological parameters for each basin. TN = Total Nitrogen; TP = Total Phosphorous; Turb = Turbidity; Cond = Conductivity. The number of sample sites for each basin are in parentheses.

	Paraná (6)	Pantanal (9)	Araguaia (13)	Amazon (10)
Secchi (m)	0.65(±0.54)	0.51(±0.14)	0.49(±0.15)	0.41(±0.35)
Turb (NTU)	42.50(±39.35)	19.79(±16.09)	31.40(±20.25)	78.01(±75.87)
pH	6.62(±0.29)	6.81(±0.50)	6.89(±0.26)	6.31(±1.08)
Cond (μS/cm)	30.18(±15.19)	83.99(±47.06)	38.38(±7.78)	75.76(±64.49)
TN (μg/L)	1483.1(±742.29)	1075.7(±69.55)	1287.8(±313.64)	2597.9(±1705.44)
NO_3_^-^ (μg/L)	21.47(±52.58)	37.46(±57.90)	24.10(±69.11)	46.47(±79.32)
NH_4+_ (μg/L)	43.83(±28.84)	28.59(±20.28)	28.43(±31.31)	44.41(±78.45)
TP (μg/L)	63.73(±36.69)	52.26(±16.56)	85.63(±29.10)	113.27(±50.75)
PO_4_^3-^ (μg/L)	17.03(±10.43)	14.52(±6.53)	12.69(±4.61)	15.45(±8.18)

Water samples were stored on ice and taken to the nearest field station, where they were filtered through GF 52-C membranes (<10 hours after sampling) and immediately frozen (–20°C) for subsequent analyses of dissolved nutrients. Water was also frozen at –20°C before filtering to measure total nitrogen and total phosphorus. Total nitrogen was analyzed with the persulfate method with oxidations of all nitrogenous compounds to N-nitrate [73]. This ion was determined in a spectrophotometer after reducing N-nitrite in the presence of cadmium, using a flow-injection system [74]. N-ammonium was quantified by the indophenol blue method, also read in a spectrophotometer [75]. Total and reactive dissolved phosphorous (i.e. P) were measured in a spectrophotometer, according to Golterman *et al*. ([76]).

Primary carbon sources collected in each lake for this study included periphyton (attached algae, mostly filamentous algae), phytoplankton, particulate organic carbon (POC), C_3_ aquatic macrophytes, C_4_ plants (aquatic and terrestrial grasses), and C_3_ riparian vegetation. Three to five replicate samples of each carbon source were collected from each site (i.e. each lake), where available. Periphyton was obtained by gently scraping the stem of aquatic plants and other submerged structure (e.g. woody debris), and was stored in a bottle with distilled water for subsequent filtering. Phytoplankton was sampled in the littoral and limnetic zones of each site using a 15 μm plankton net horizontally dragged twice in each zone, constituting four samples per lake. POC was obtained by filtering water collected directly from the subsurface of littoral and limnetic zones in each lake. Periphyton samples and samples of water containing phytoplankton and POC were individually filtered and retained on pre-combusted (400°C for 4 hours) 47mm glass fiber filters (Whatman GF/C). Aquatic macrophytes (i.e. emergent and floating plants), riparian vegetation and C_4_ plants consisted of multiple leaves of the most common and abundant vascular plants in each sample site, clipped directly from the plant, and separated by species. The replicates of plant leaves were composed by one leaf of one plant, i.e. in each lake we sampled 3–5 different plants, each one constituting one replicate.

All samples were dried in an oven at 60°C for 72h hours and macerated to obtain a fine and homogeneous powder using a ball-mill grinder or mortar and pestle. Sub-samples of approximately 3–4 mg for C_3_ riparian vegetation, C_4_ plants and aquatic macrophytes, or half of a filter containing phytoplankton, periphyton or POC were pressed into tin capsules (Costech Analytical, CA, USA) and sent to the University of California at Davis Stable Isotope Facility (USA) for determination of carbon and nitrogen isotope ratios. Results are expressed in delta notation (parts per thousand deviation from a standard material): δ^13^C or δ^15^N = [(*R*_*sample*_/*R*_*standard*_) -1] *1000; where *R* = ^13^C/^12^C or ^15^N/^14^N. The standard material for carbon is Vienna Pee Dee Belemnite (V-PDB) limestone, and the nitrogen standard is atmospheric nitrogen. Standard deviations of δ^13^C and δ^15^N for five different replicate analyses of internal standards were between 0.04‰ and 0.13‰ and 0.09 ‰ and 0.22 ‰, respectively.

Subsequent analyses are based on carbon and nitrogen values for 606 samples across all basins and carbon sources. It is important to mention that aquatic macrophytes were absent in the Araguaia floodplain during the sampling period, and thus are not included in our dataset and analyses. Low sample sizes for C_4_ plants from the Paraná and Araguaia floodplains (i.e. one in each system) are not expected to bias our analyses of δ^13^C since the values of this source have low variation among ecosystems (e.g. [8]), but would likely change the results for δ^15^N analyses because δ^15^N of plants is more dependent on nutrient loads of the ecosystem than the photosynthetic pathway of the plant.

### Data analysis

In order to answer our two primary questions, we performed a series of analyses with stable isotope values as response variables, and limnological conditions and basin identity as predictors. First, a principal coordinate analysis (PCoA) was performed to reduce the dimensionality of the limnological data, and the broken-stick criteria [77] was used to determine the relevant number of axes for interpretation. Multivariate analysis of variance (MANOVA) was subsequently applied using PC scores of the retained axes to test for differences in limnological conditions among basins. Analysis of variance (ANOVA) was used to test for differences in isotope values (separately for δ^13^C and δ^15^N) of each source among basins, with a Tukey HSD posthoc test for distinguishing pairwise relationships among basins following a significant main effect. Pearson correlations were performed between all limnological parameters as well as between those parameters and source isotope values (δ^13^C and δ^15^N).

Regression trees were used to predict isotope values (separately for δ^13^C and δ^15^N) based on limnological parameters and basin identity (i.e. surrogate for geologic formation). Classification and regression trees are powerful nonparametric approaches to modeling complex ecological data and provide a flexible alternative to linear and additive models [78]. Tree generation involves successively partitioning the response variable into increasingly homogeneous subsets based on fit with predictor variables, including an ability to identify and express non-linear, nested and non-additive relationships. This is particularly appealing in situations where hierarchical interactions are present, and relationships between the response variable and some predictor variables are conditional on the values of other predictors [79]. We used the Gini index to minimize impurity of non-parent nodes, the minimum number of observations at a node in order for a split to be attempted (*minsplit*) was set at 10, and maximum tree depth (*maxdepth*) was set at four. The optimum size of the regression trees (*pruning*) was determined by selecting the tree size with the smallest model error based on repeated cross-validation of the data.

All analyses were conducted using R [80]. Specifically, regression trees were performed using the package Rpart [81] and the other analyses were performed using the packages Stats [80] and Vegan [82]. Significance of statistical tests was assessed at α = 0.05.

## Results

**Fig 2:**
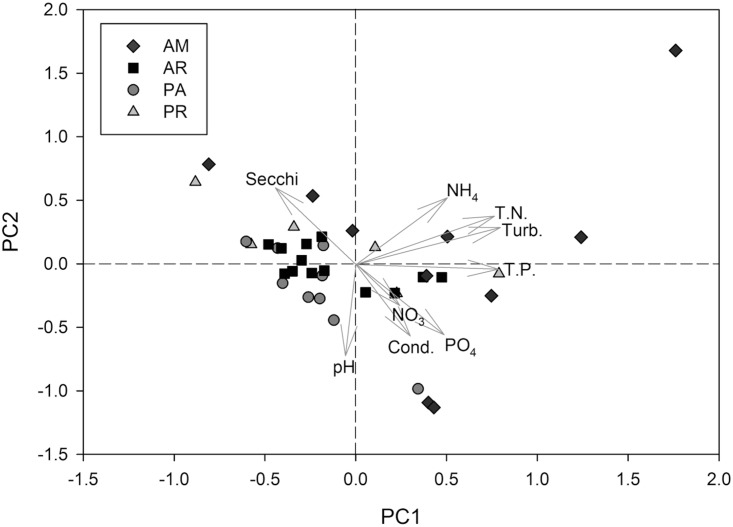
Principle coordinates analysis biplot of limnological conditions among sampling locations and basins. The first two axes explain 59% of the variation among sites (i.e. PC1 explained 36% and 23% was explained by PC2). AM = Amazon; AR = Araguaia; PA = Pantanal; PR = Paraná.

The first two PCoA axes explained 59% of the variation in limnological conditions among sites (Fig 2). The first axis separated the sample sites based on turbidity and nutrient concentrations (primarily TN and TP) at positive values, and water clarity (i.e. Secchi depth) at negative values. Secchi depth and ammonia concentration were associated with positive values on PC2, whereas pH, conductivity and phosphate concentration were the primary variables associated with negative values on PC2. Although sampling locations in Amazon had, on average, higher nutrient concentrations and turbidity (Table 1), substantial within basin heterogeneity resulted in broadly overlapping distributions of basins in the PCoA and a non-significant MANOVA (Fig 2; Pillai = 0.32; *F*_*3*,*34*_ = 2.14; *p* = 0.06). Similar to the pattern of axis loadings in the PCoA, several limnological parameters were highly correlated in pairwise comparisons (S1 Fig). Specifically, strong positive correlations were observed among turbidity, TN, TP and NH_4_^+^, and between pH and conductivity, whereas Secchi depth was negatively correlated with TP and PO_4_^3-^.

No significant differences were observed for δ^13^C values of carbon sources among basins, though high within-basin variability was observed for periphyton, phytoplankton and POC (Table 2). In contrast, significant differences in δ^15^N were observed among basins for all carbon sources except C_4_ plants (Table 2). Sources from the Araguaia floodplain were consistently more ^15^N-enriched than in the other basins (Table 2), and exhibited significant differences between the Pantanal for C_3_ riparian vegetation, the Paraná floodplain for periphyton and phytoplankton, and among all other basins for POC. C_3_ macrophytes had significantly higher δ^15^N in the Pantanal than Paraná (Table 2). δ^15^N values for sources from the Amazon were intermediate in all cases.

**Table 2:** Mean (‰) and standard deviation (±SD) of carbon and nitrogen isotope values for each carbon source, and ANOVA results for the test of differences among basins. PR = Paraná; PA = Pantanal; AR = Araguaia; AM = Amazon; n = number of samples for each source in each basin. Shared superscript lowercase letters indicate lack of significant differences for the Tukey post-hoc test.

	PR	PA	AR	AM	ANOVA		
	Mean (±SD)				F_df_	*p*	Tukey test
**Periphyton (n)**	**12**	**17**	**20**	**7**			
δ^13^C	-29.03 (1.82)	-26.26 (5.42)	-27.21 (2.55)	-25.03 (0.78)	F_3,52_ = 2.30	0.088	-
δ^15^N	4.72 (1.69)	5.87 (1.20)	6.22 (1.49)	5.17 (0.83)	F_3,52_ = 3.31	**0.027**	PR^a^ AM^a,b^ PA^a,b^ AR^b^
**Phytoplankton (n)**	**25**	**36**	**52**	**36**			
δ^13^C	-30.10 (3.07)	-30.83 (3.79)	-30.19 (1.93)	-30.12 (1.94)	F_3,145_ = 0.59	0.621	-
δ^15^N	3.96 (2.30)	5.01 (1.49)	5.42 (1.25)	4.67 (1.65)	F_3,145_ = 4.90	**0.003**	PR^a^ AM^a,b^ PA^b^ AR^b^
**P.O.C. (n)**	**22**	**36**	**53**	**40**			
δ^13^C	-29.87 (2.55)	-30.39 (3.80)	-30.64 (1.72)	-30.67 (2.03)	F_3,147_ = 0.59	0.626	-
δ^15^N	3.94 (1.83)	4.19 (1.54)	5.10 (1.32)	4.27 (1.19)	F_3,147_ = 5.24	**0.002**	PR^a^ PA^a^ AM^a^ AR^b^
**C**_**3**_ **Macrophytes (n)**	**19**	**38**	**0**	**10**			
δ^13^C	-29.49 (0.85)	-29.05 (1.36)	-	-29.62 (1.15)	F_2,64_ = 1.34	0.270	-
δ^15^N	4.86 (2.41)	7.54 (2.17)	-	5.53 (0.49)	F_2,64_ = 10.61	**<0.001**	PR^a^ AM^a,b^ PA^b^
**C**_**4**_ **Plants (n)**	**1**	**13**	**1**	**10**			
δ^13^C	-12.82	-12.49(0.67)	-12.56	-12.60 (1.02)	F_3,21_ = 0.07	0.976	-
δ^15^N	6.20	5.21(2.64)	9.92	6.43 (1.43)	F_3,21_ = 1.74	0.191	-
**C**_**3**_ **Riparian Vegetation (n)**	**25**	**42**	**54**	**37**			
δ^13^C	-29.86 (1.46)	-30.04 (1.20)	-30.08 (1.16)	-30.50 (0.94)	F_3,154_ = 1.74	0.162	-
δ^15^N	4.64 (2.68)	3.12 (3.06)	4.65 (2.30)	3.68 (1.47)	F_3,154_ = 3.91	**0.010**	PA^a^ AM^a,b^ PR^a,b^ AR^b^

All significant correlations between δ^13^C or δ^15^N and limnological parameters were relatively weak (i.e. | *r* | < 0.42), and, when significant, were predominantly observed for phytoplankton, POC and periphyton (Table 3). δ^13^C values for phytoplankton and POC were positively correlated with turbidity, TP and PO_4_^3-^ (plus TN for phytoplankton), and negatively correlated with Secchi depth. Periphyton δ^13^C was negatively correlated with NH_4_^+^. In contrast, δ^15^N values for phytoplankton and POC were positively correlated with pH and NO_3_^-^, and negatively correlated with turbidity, TN and NH_4_^+^ (plus PO_4_^3-^ for POC). Periphyton δ^15^N was positively correlated with NO_3_^-^ and NH_4_ and negatively correlated with turbidity and PO_4_^3-^. C_3_ aquatic macrophyte δ^15^N was negatively correlated with turbidity. No environmental correlates of δ^13^C or δ^15^N were found for C_4_ plants or C_3_ riparian vegetation (except for a very weak positive correlation between turbidity and δ^13^C).

**Table 3:** Pearson correlation coefficients between stable isotope values (δ^13^C or δ^15^N) and environmental variables for each source. Significant values are in bold with level of significance denoted using asterisks (* *p*<0.05; ** *p*<0.01; *** *p*<0.001). Turb = Turbitity, Cond = Conductivity, TN = Total Nitrogen, TP = Total Phosphorous.

	Secchi	Turb.	pH	Cond.	TN	NO_3_^-^	NH_4_^+^	TP	PO_4_^3-^
**Periphyton**									
δ^13^C	-0.210	-0.067	-0.070	0.119	0.038	-0.094	-0.312*	0.101	0.107
δ^15^N	0.173	-0.273*	0.119	0.074	-0.112	0.419**	0.329*	0.030	-0.286*
**Phytoplankton**									
δ^13^C	-0.333***	0.320***	-0.110	0.012	0.187*	-0.034	0.002	0.281***	0.366***
δ^15^N	-0.016	-0.285***	0.259**	0.067	-0.214**	0.260**	-0.212**	-0.044	-0.142
**P.O.C.**									
δ^13^C	-0.235**	0.227**	0.044	0.134	0.121	-0.095	-0.064	0.182*	0.342***
δ^15^N	0.023	-0.305***	0.251**	-0.010	-0.204*	0.276***	-0.183**	-0.009	-0.193*
**C**_**3**_ **Macrophytes**									
δ^13^C	0.089	-0.141	-0.030	0.011	-0.216	-0.141	-0.121	-0.210	-0.133
δ^15^N	-0.075	-0.309*	-0.140	0.045	-0.156	0.071	0.063	-0.078	0.063
**C**_**4**_ **Plants**									
δ^13^C	0.034	-0.045	0.056	-0.068	-0.239	0.257	0.008	-0.151	0.070
δ^15^N	-0.019	0.159	0.011	-0.192	0.159	-0.137	-0.138	0.256	0.103
**C**_**3**_ **Riparian Vegetation**									
δ^13^C	-0.174	0.061*	0.051	0.004	0.049	-0.077	0.095	0.075	0.114
δ^15^N	-0.041	-0.041	0.125	0.034	-0.055	0.051	0.006	0.107	0.000

**Fig 3:**
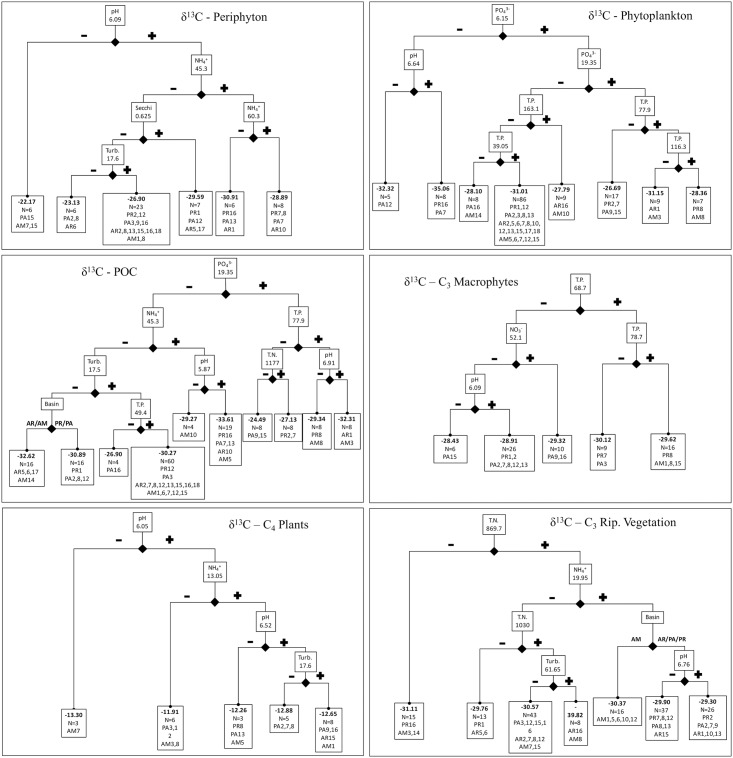
Regression trees predicting δ^13^C for each carbon source. Splits are identified by boxes on branches with the corresponding parameter and value, and terminal nodes are identified by boxes including the mean value for the response variable (i.e. δ^13^C) as well as the number of samples and sampling locations included in the node. Plus and minus signs or basin abbreviations designate the level of parameter to the left or right of a split (e.g. pH less than or greater than 6.09 for the first split for periphyton).

**Fig 4:**
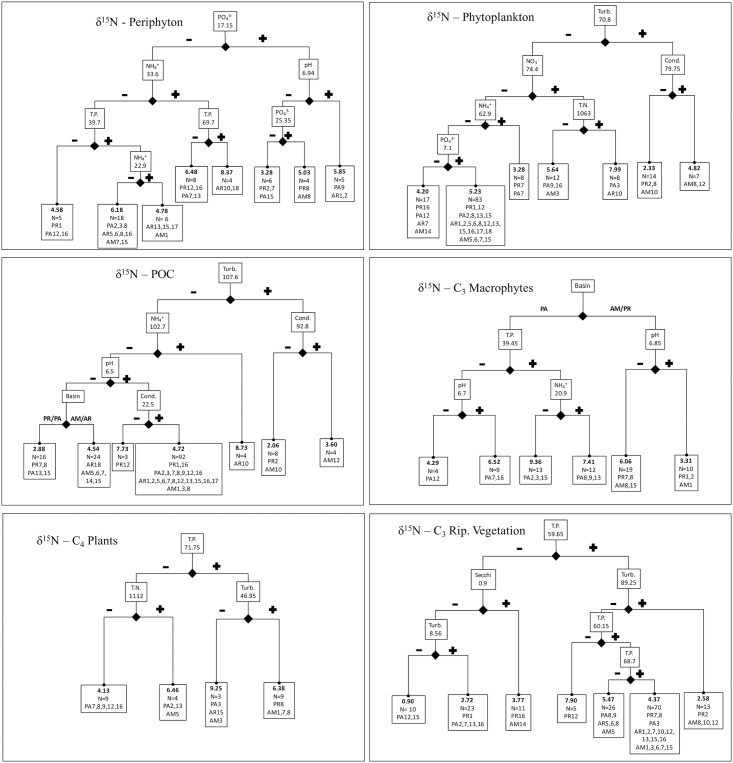
Regression trees predicting δ^15^N for each carbon source. Splits are identified by boxes on branches with the corresponding parameter and value, and terminal nodes are identified by boxes including the mean value for the response variable (i.e. δ^15^N) as well as the number of samples and sampling locations included in the node. Plus and minus signs or basin abbreviations designate the level of parameter to the left or right of a split (e.g. TP less than or greater than 59.65 for the first split for riparian vegetation).

Regression trees for both δ^13^C and δ^15^N depict complex multivariate and in some cases nested relationships with local limnological conditions and basin identity (Figs 3 and 4). All of the measured limnological parameters and basin identity were included in at least two regression trees, but TP, pH, NH_4_^+^ and turbidity together accounted for almost 70% of the splits (including the primary split in seven of the 12 regression trees; Figs 3 and 4). Although PO_4_^3-^ was included in only four models, three of those were primary splits (i.e. δ^13^C of phytoplankton and POC, and periphyton δ^15^N). Similarly, basin identity was included in four models but was the primary split for only δ^15^N of C_3_ aquatic macrophytes, distinguishing samples from the Pantanal from low- and high-pH sites in the Paraná and Amazon floodplains (subsequent splits for the Pantanal were associated with TP, pH and NH_4_^+^; Fig 4). Aside from C_3_ macrophytes from the Pantanal, almost all other terminal nodes across all trees were comprised by samples from more than one basin (Figs 3 and 4).

Tree structures for δ^13^C and δ^15^N were never identical for a carbon source, and exhibited limited similarity across sources for δ^13^C and δ^15^N. Coarsely comparing models for δ^13^C and δ^15^N, model complexity was similar (i.e. similar number of splits), but the importance of a few parameters were more commonly associated with one or the other element. Specifically, turbidity was included four and five times in regression trees for δ^13^C and δ^15^N, respectively, but was never a primary or secondary split for δ^13^C whereas four of the five inclusions for δ^15^N were either primary or secondary splits (Figs 3 and 4). Seemingly linked to high turbidity, conductivity was a secondary split for the two δ^15^N trees with turbidity as the primary split (i.e. phytoplankton and POC), but was never included in δ^13^C models. pH was included twice as many times in regression trees for δ^13^C, including two primary and one secondary split (i.e. C_4_ plants, periphyton and phytoplankton) versus two secondary splits for δ^15^N (i.e. C_3_ macrophytes and periphyton). Nutrient concentrations were frequently included and in similar numbers in trees for both δ^13^C and δ^15^N. However, N (i.e. TN, NO_3_^-^, NH_4_^+^) was mostly a secondary split (nine secondary splits, one primary) whereas P (i.e. TP and PO_4_^3-^) was commonly included as a primary and secondary split (six primary, five secondary).

## Discussion

Previous studies demonstrated biogeographic (i.e. among systems) and biochemical (i.e. within systems) effects on carbon and nitrogen stable isotope values (e.g. [27, 83, 84]). In this study, only very minor differences in δ^15^N values of sources were observed among basins, and δ^13^C did not differ among basins for any source (although high within-basin variability was observed for periphyton, phytoplankton and POC, likely precluding significant differences among basins). Although δ^13^C and δ^15^N values exhibited significant correlations with some limnological factors within and among basins, those relationships differed among carbon sources.

When considering basin identity and local limnological conditions together, regression trees for both δ^13^C and δ^15^N for all sources depicted complex and in some cases nested relationships, and only very limited similarity was observed among trees for different carbon sources. Contrary to expectations, nutrient concentrations did not have a consistent directional effect on isotope values (e.g. positive correlation between δ^15^N and nitrogen concentration). That being said, some factors were more consistently included in regression trees for δ^13^C or δ^15^N and at primary or secondary split locations (i.e. more important in determining isotope values). For example, turbidity was generally more important for δ^15^N (two primary and two secondary splits), whereas pH was included twice as many times in regression trees for δ^13^C (including two primary and one secondary split). Nutrient concentrations were frequently included and in similar numbers in trees for both δ^13^C and δ^15^N, but N (i.e. TN, NO_3_^-^, NH_4_^+^) was mostly a secondary split (nine secondary splits, one primary) whereas P (i.e. TP and PO_4_^3-^) was commonly included as a primary and secondary split (six primary, five secondary). Although limnological conditions and basin identity predicted variation in isotope values of carbon sources, in our opinion the resulting models are too complex to provide a reasonable platform for mathematical correction or alignment of isotope values among sites (e.g. source corrections for evaluation of river ecosystem models).

In regards to our second question, our findings indicate a more pronounced effect of local limnological conditions on variation in δ^13^C and δ^15^N of basal sources, regardless of floodplain-system (i.e. basin identity). Basin was the primary factor affecting δ^15^N of C_3_ macrophytes (which generally have high values for δ^15^N in Pantanal; [85]) but nutrient concentrations and pH were important in further distinguishing the broad range of variability in δ^15^N of macrophytes in the Pantanal. In contrast, Jepsen and Winemiller ([27]), working in four tributaries of the Orinoco River basin, identified basin geochemistry as a primary factor affecting δ^13^C and δ^15^N values of sources and consumers (i.e. fishes). In their study, the tributaries exhibited general differences in limnological conditions due to underlying geologic formations (i.e. white, clear and black water types). Significant variability in δ^13^C and δ^15^N of sources was observed within each system (i.e. distribution of source values within systems exceeded differences among systems in their Fig 4), but their primary focus was on upper trophic levels and they did not investigate factors affecting isotopic variability of sources within each system. We expect that had they further explored factors affecting source variability among sampling locations, the effect of ‘basin’ on the isotopic baseline would have been greatly diminished. However, that leaves the question of why consumers demonstrated marked differences in isotope values among systems. This is likely due to differences in the relative importance of various sources to the food webs (as discussed by the authors), which would compound any differences in the mean isotope values of specific sources among systems. In comparison, our study lacked the range of limnological conditions (i.e. extreme blackwater) in Jepsen and Winemiller ([27]), and we would anticipate a greater influence of basin geochemistry on source δ^13^C and δ^15^N had our study included a classic blackwater river (e.g. the Rio Negro in the Amazon basin).

No significant differences in δ^13^C of carbon sources were observed at the landscape scale, i.e. comparing mean values of sources among floodplains, and mean source values were comparable with previous studies in floodplain ecosystems (e.g. [8, 46, 83, 86]). Although largely conserved by photosynthetic pathway (e.g. C_4_ vs. C_3_ plants) and by assimilation of atmospheric CO_2_ for vascular plants, regression trees identified complex relationships between δ^13^C of carbon sources and local limnological conditions. Somewhat surprisingly, pH was associated with the primary split in δ^13^C regression trees in only two cases (periphyton and C_4_ plants). pH is one of the distinguishing characteristics in the classification of water types that previous studies have associated with differences in δ^13^C (e.g. [27]). It also plays a fundamental role in the bicarbonate equilibrium (i.e. determining relative concentrations of dissolved inorganic carbon compounds) and affects δ^13^C of DIC [19–21]. After pH, the subsequent split was associated with NH_4_^+^, perhaps indicating an important interaction between nutrient concentrations and pH in determining δ^13^C of the DIC [22, 23] and subsequently δ^13^C of periphyton. For C_4_ plants, this is probably a spurious result because the main source of carbon for vascular plants is atmospheric CO_2_ [29]. However, pH was included in every δ^13^C regression tree, and was always in combination with nutrient concentrations for autochthonous sources. Autochthonous production sources such as phytoplankton preferentially assimilate dissolved ^12^CO_2_ during photosynthesis, thus the rate of photosynthesis (often limited by available nutrients and light) can affect δ^13^C of the DIC by ^12^C depletion.

It has long been recognized that lake metabolism plays an important role in influencing the isotope signature of DIC [87]. For example, increasing productivity increases δ^13^C-DIC [88] and respiration is generally considered to be the reason for declining δ^13^C-DIC [89]. Although Bade *et al*. ([21]) found a weak correlation between TP and δ^13^C-DIC and a strong effect of pH on δ^13^C-DIC for the Highland Lakes (USA), this pattern may be different in highly productive floodplain ecosystems [46]. δ^13^C of autochthonous carbon sources (i.e. phytoplankton, periphyton) are directly dependent on DIC in floodplain-river systems and seem to be governed by a balance between respiration and productivity due to a greater contribution of biogenic CO_2_ in these productive watersheds [90]. Unfortunately, we were unable to process samples for determination of δ^13^C-DIC, which would have allowed us to more directly test for relationships between limnological conditions and δ^13^C of DIC and primary production sources among sampling locations.

δ^15^N of carbon sources was expected to increase with increasing nutrient concentrations (e.g. [32, 91]). In contrast, our correlation analyses indicated no relationship between δ^15^N and nutrient concentrations for some sources as well as both positive and negative correlations between δ^15^N and different nutrient species (e.g. NH_4_^+^ vs NO_3_^-^) for the same carbon source. Although nutrient concentrations were frequently included in the regression trees, there was not a consistent pattern of higher δ^15^N values with higher nutrient concentrations and all models included other predictor variables not associated with nutrients (e.g. pH, conductivity, basin identity). Thus, the influence of nutrient availability on δ^15^N of carbon sources was dependent on other factors and was not consistent among sources. Pollution from anthropogenic activities, such as agriculture (e.g. [38, 92]) and sewage [39, 93] have been shown to affect δ^15^N of primary producers and consumers. Although we did not directly incorporate sampling to assess such human activities in this study, we observed enriched δ^15^N values of phytoplankton (4.46‰) in the Amazon lake with the highest TN and TP concentrations across all sites (i.e. site AM12, surrounded by an active human community).

It is important to note that there are other potentially important environmental factors that we did not quantify. Specifically, gradients in δ^13^C and δ^15^N of algae have been attributed to water velocity [14, 94], high temperatures and light intensity [95, 96]. Our study sites were all located in floodplain lakes with little or no flow, consistently high temperatures (i.e. > 25°C) and open canopy, so inclusion of those additional parameters is unlikely to significantly change our findings. However, seasonal variation may influence stable isotopes values of carbon sources. For example, Cloern *et al*. ([97]) observed seasonal shifts in carbon and nitrogen isotope values of wetland plants from an estuarine system, which they attributed to species-specific cycles of plant growth and senescence. Freshwater floodplain systems are as complex as estuarine systems, however the primary driving force that accounts for seasonal variation in limnological conditions is the flood pulse [2, 98]. Nutrients from different sources (e.g. main river, sediment, decomposing vegetation; [99]) flow into marginal lakes during the rainy season which can directly influence stable isotope values of aquatic primary producers [21, 100]. At the same time, the flood pulse tends to homogenize limnological conditions across the floodplain [72], therefore the high within floodplain heterogeneity observed in our dry season samples would not be expected during the rainy season.

Landscape-level differences in stable isotope values of production sources have been useful in studies of organism movement [50, 101–103]. For example, differences in δ^13^C of sources of white and black water rivers discussed above allowed Winemiller and Jepsen ([49]) to estimate subsidies to blackwater food webs via consumption of migratory *Semaprochilodus kneri* by large peacock bass *Cichla temensis* [104]. For our study systems, high *within* floodplain variability during the dry season and importance of local conditions in determining those differences, suggest that isotopes may be useful for examining habitat use, dispersal and patch dynamics within heterogeneous floodplain ecosystems rather than just between systems with extreme water types. If validated, this approach could represent a finer scale patch or mesohabitat application than previously utilized ‘isoscape’ approaches [51, 105–107]. Such an application would require relatively consistent differences in basal sources among patches and would likely be more useful for small-bodied consumers or early life stages of larger-bodied species (e.g. young-of-year fishes) that rely on algal sources (due to greater variability in those sources and faster tissue turnover rates in smaller-bodied consumers). In addition to small-bodied species or life stages, specific tissues with relatively fast turnover rates, such as liver and blood, could also be utilized. Movement and dispersal rates are notoriously difficult to quantify in complex and open systems such as floodplains, and the application of natural isotopic tracers would be a welcome addition to the ‘tool kit’ for such studies where artificial isotopic labeling (e.g. [34, 108, 109] is not feasible due to the spatial scale and size of the water bodies. The inclusion of hydrogen stable isotopes along with carbon and nitrogen may further enhance this possibility [110, 111], similar to the utility of sulfur stable isotopes for adding greater resolution along spatial gradients in coastal systems [112, 113], and complement otolith microchemistry (e.g. [114]).

The same heterogeneity that may enable the use of isotopes as tracers of organism movement may complicate their use in other types of investigations. One of our primary interests in understanding factors that affect variation in isotope values of basal carbon sources was to identify when and how that variability may need to be accounted for when comparing isotope values of consumers from across sampling locations or systems. For example, understanding and potentially accounting for such baseline variation is important for testing the aforementioned models of carbon flow in river ecosystems (e.g. [8, 9, 11]). Our findings paint a complex picture of the effects of local conditions versus landscape differences on the carbon and nitrogen isotope values of production sources. At the landscape scale, δ^13^C of different carbon sources was relatively conserved across systems, which is good news for comparing patterns of carbon flow using this tracer. However, δ^15^N differed among systems as well as among sources within systems (in some cases more than the expected effect of trophic fractionation) which is a significant concern when working with upper trophic level taxa or multitrophic assemblages. Unfortunately, the complex interactions of multiple factors in determining isotope values of sources among sites likely precludes a simple analytical baseline correction. Such variability in the δ^15^N baseline is often accounted for by using relatively large-bodied primary consumers as indicators of baseline values [16, 18, 115]. The relatively greater heterogeneity in source isotope values within versus among systems in our study highlights that the spatial scale of sampling, connectivity among patches and habitat use of potential baseline indicator taxa should be explicitly considered.

## Supporting information

S1 Fig Pearson correlation matrix of limnological parameters. Coefficients are provided above the diagonal and raw values are provided below the diagonal. Asterisks designate significance level (*p<0.05, **p<0.01, ***p<0.001). Turb = Turbidity, Cond = Conductivity, TN = Total Nitrogen, TP = Total Phosphorous.
(PDF)

S1 Spreadsheet Raw carbon and nitrogen stable isotopes data. Data from of all six carbon sources (i.e. periphyton, phytoplankton, POC, C3 macrophytes, riparian vegetation, and C4 plants) sampled across four Neotropical floodplains (Paraná, Pantanal, Araguaia, and Amazon).
(XLSX)
