# Supplementary material for: Dry season limnological conditions and basin geology exhibit complex relationships with δ13C and δ15N of carbon sources in four Neotropical floodplains
Source: PLoS One. 2017 Mar 30;12(3):e0174499. doi: 10.1371/journal.pone.0174499 (PMC5373579; doi:10.1371/journal.pone.0174499)

Pearson Correlation Matrix

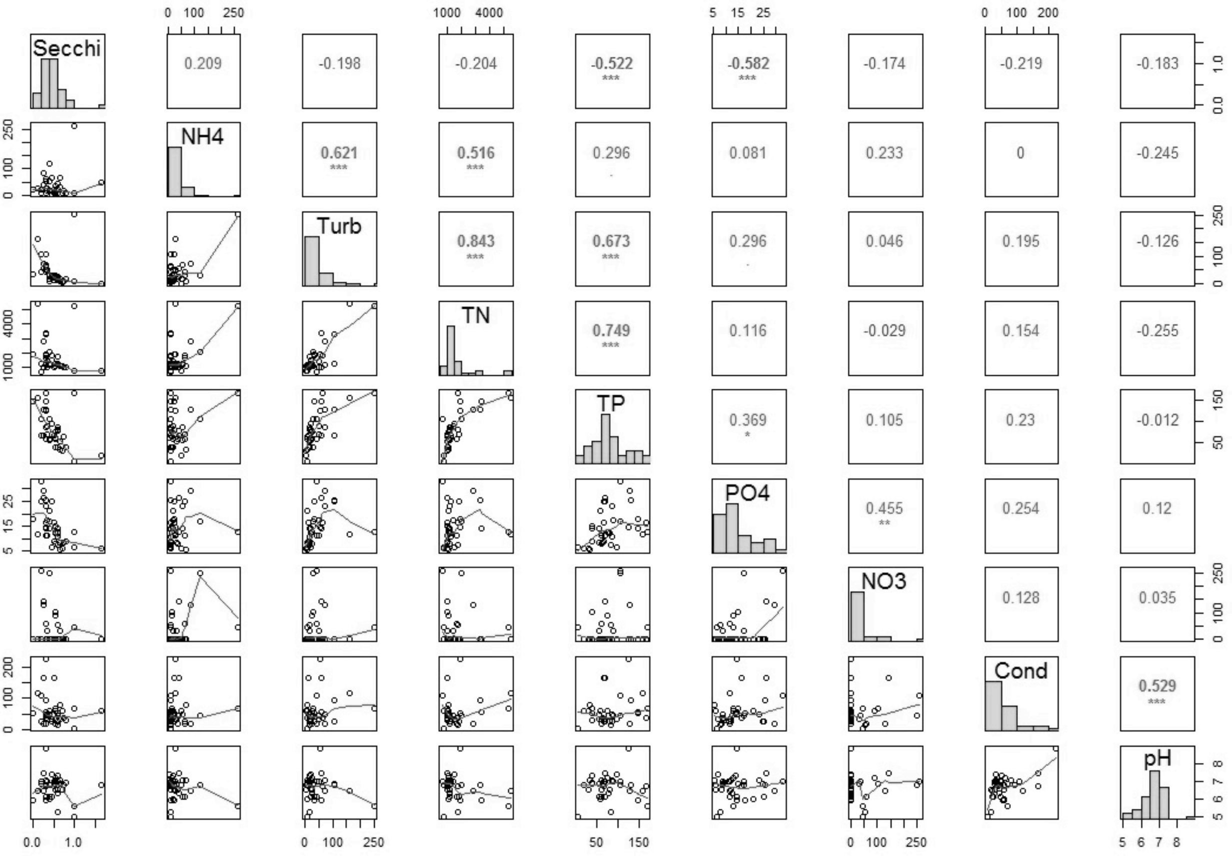

Supplement: S1 Fig — Coefficients are provided above the diagonal and raw values are provided below the diagonal. Asterisks designate significance level (*p<0.05, **p<0.01, ***p<0.001). Turb = Turbidity, Cond = Conductivity, TN = Total Nitrogen, TP = Total Phosphorous. (PDF) [file pone.0174499.s001.pdf]
